# Riding on the crest of electronic publishing wave

**DOI:** 10.2349/biij.5.1.e1

**Published:** 2009-01-01

**Authors:** NA Kadri, LK Tan, KH Ng

**Affiliations:** 1 Department of Biomedical Engineering, Faculty of Engineering, University of Malaya, Kuala Lumpur, Malaysia; 2 Department of Biomedical Imaging, Faculty of Medicine, University of Malaya, Kuala Lumpur, Malaysia

## THE STORY SO FAR

When biij came into being in early 2005, it was envisioned as a tool for the dissemination of scientific knowledge. Today, biij continues to fulfill this role, having gone through a process of evolution and growth.

In the early days [1], the email system was the main means of communication. All manuscript submissions, including its figures and images, were sent as email attachments to the managing editor. The subsequent reviewing stages were also done via email. To simplify the tracking process, the managing editor developed an in-house software to view and update the status of each of the submitted manuscripts. It was developed using the Active Server Pages (ASP) programming language and a Microsoft Access database, and is only viewable by the editors. Although the software served its purpose well, the journal has grown so much so that its requirements for additional features far outweigh the resources that were currently available.

From January 2007, biij gradually implemented the open source Open Journal Systems (OJS) software for online manuscript submission, tracking and management. The software was developed as part of the Public Knowledge Project, managed in partnership between the Faculty of Education at the University of British Columbia, the Simon Fraser University Library, the School of Education at Stanford University, and the Canadian Centre for Studies in Publishing at Simon Fraser University [2].

From the outset of biij's birth, the publishers have always emphasised the searchability and availability of biij’s contents. In October 2005, biij became a member of CrossRef [3] and provided a unique Digital Object Identifier (DOI) for each of the published manuscripts, including the abstracts from selected meetings and conferences. This ensures that biij’s contents remain available in the future, even if there are changes to the structure of its website.

Biij is now indexed in a number of indexing databases, including Scopus, Embase, and Compendex (since January 2008); Chemical Abstracts Service (CAS) (since April 2006); INSPEC (since March 2006); Index Copernicus International (since April 2006); Google Scholar (since December 2005); and Directory of Open Access Journals (DOAJ) (since September 2005). In November 2008, Elsevier also agreed to include all manuscripts prior to 2008 in its Scopus and EMBASE database.

Biij contents are currently being submitted to Pubmed Central [4], the online repository for biomedical and life sciences journal literature at the U.S. National Institutes of Health (NIH) [5]. This process is part of the requirements for the application of evaluation for the Pubmed/Medline database, to be initiated sometime in 2009.

## THE FUTURE AND BEYOND

The main objective of a journal is the dissemination of scientific knowledge. The concept of biij was to offer a platform for effective communication between the authors and readers [1]. One jargon that has been used is ‘enhanced discourse’. It refers to an expanded and facilitated scientific discourse about research, online letters to the editor and discussions concerning articles with links to the articles in question.

To achieve the above, a new feature was introduced recently, a feedback/ comment box where readers can send their comments to the journal. This feature, hopefully, will enhance the communication between the authors and the scientific community.

There are, of course, challenges that lie ahead. For a biomedical imaging journal, high quality images play a prominent part in the manuscripts. However, imaging journals still publish images in low resolution formats, such as jpeg. Until today, there is still no appropriate interface for readers to display and interact with DICOM images in real time while reading the paper. Many researchers and readers recognise that there is a need to have a browser-based image viewer embedded into the journal in a seamless manner.

The other challenge is to maximise the multimedia features of biij. One of the biggest attractions for journals to go electronic is the potential utilisation of multimedia in the paper – for example, the inclusion of video clips, 3D movies, animation, etc.

The journal *Medical Physics* is using the Electronic Physics Auxiliary Publication Service (EPAPS) [6]. It is an electronic depository for material that is supplementary to papers appearing in journals published by or through the American Institute of Physics (AIP). Appropriate items for deposit include multimedia (e.g., movie files, audio files, animated .gifs, 3D rendering files), colour figures, data tables, and text (e.g., appendices) that are too lengthy or of too limited interest for inclusion in the printed journal. Materials are available free of charge to users via links from the online journals or by browsing the EPAPS' depository directories.

However, not many biij authors have taken advantage of this multimedia feature. One of the reasons could be due to the technical hassles involved. It may take several more years for this feature to become the norm.

As Internet technology is progressing ever so rapidly, the landscape of electronic publishing will be continually changing as well. The world is witnessing a revolutionary change in the paradigm of scholarly publishing.
